# Preoperative Urine Testing in Elective Endourology Procedures: Is a Change in Practice the Need of the Hour?

**DOI:** 10.7759/cureus.98888

**Published:** 2025-12-10

**Authors:** Chithra Sugathan Sheela, Rebecca Saunders

**Affiliations:** 1 Urology, Royal Gwent Hospital, Newport, GBR

**Keywords:** complications, endourology, post op infection, urine culture, urine testing

## Abstract

Introduction

Urine testing is routinely done preoperatively in patients undergoing elective endourology procedures to ensure that any asymptomatic bacteriuria is picked up and treated. This practice, in addition to leading to overtreatment, can also lead to unnecessary cancellations of elective procedures. With the emergence of multidrug-resistant bacteria and the implementation of stricter antibiotic stewardship programmes, it is the need of the hour to re-examine this innocuous practice.

Patients and methods

One hundred patients undergoing elective endourology surgery were studied retrospectively to assess current practice at a district and general hospital in Wales, UK. All patients received a single dose of intraoperative antibiotic at induction. These patients were followed up for two weeks to look for postoperative septic complications.

Results

Of the 100 patients, 15% had cultures done in the stipulated 2-week period preceding surgery. Seven patients (7%) developed postoperative UTIs, of which none became acutely unwell. Of the six patients with preoperative positive cultures, none developed symptomatic UTIs post-op. Only upper tract stone surgeries were managed with a preoperative antibiotic course prior to surgery.

Conclusions

In the era of emerging antibiotic resistance and stricter antibiotic stewardship programmes, the routine practice of treating asymptomatic bacteriuria in all patients undergoing endourology surgery needs revisiting. There is an urgent need to identify the subset of patients most at risk for post-op septic complications and use targeted perioperative antibiotic prophylaxis to overcome this risk.

## Introduction

Asymptomatic bacteriuria(ASB) is defined as the presence of positive urine cultures in the absence of any symptoms of a urinary tract infection (UTI). It is common in the general population, with estimates being upto 5% in healthy premenopausal women and going up to 40-80% in those aged >80 years. Women tend to have a greater chance of ASB irrespective of age group [1]. However, a majority of this does not lead to adverse consequences. Patients with indwelling catheters are more likely to have ASB owing to colonisation of the foreign bodies by organisms. In fact, it is estimated that in patients with an indwelling catheter >28 days, 100% will grow organisms in urine [2].

It is recommended that asymptomatic bacteriuria be treated in individuals who stand to benefit the most from treatment. This includes pregnant women, patients undergoing urology procedures that breach the mucosa, and renal transplant recipients in the first three months post-transplant [1]. However, the timing of this culture and the duration of antibiotic use is not specified. French Urology guidelines recommend that the culture be done 2 weeks prior to planned surgery and ASB be treated with antibiotics 48 hrs prior to the procedure, along with a one-off antibiotic at induction [3]. Thus, the urine culture aids treatment as well as targeted prophylaxis at the time of surgery.

Positive urine cultures constituted one of the major reasons for the cancellation of operations in patients listed for Endourology procedures, according to a single-site experience from Barts Health NHS Trust. This constituted 27% of all cancelled urological operations [4]. This amounts to a waste of theatre time as well as unnecessary distress to patients due to the cancellation or postponement of surgery. This prompted a GIRFT (Get It Right the First Time) guideline in April 2025 that laid down the procedures that warranted urine testing and the timing and procedure to follow in the face of positive urine cultures [5]. 

This study was undertaken to understand current practice and identify areas where change could potentially be implemented at our institution, for the better utilisation of resources and optimum patient outcomes.

The primary objective of the study was to assess the timing of preoperative urine testing in patients undergoing Endourology procedures. 

The secondary objectives were 1. Postoperative infection rates (which were defined as readmission or prolonged hospital stay following the procedure due to a UTI); 2. Use of pre- and postoperative prophylactic antibiotics and their indications.

Patients who were readmitted or stayed longer owing to other issues that were not related to infection were not considered in this study.

## Materials and methods

This was a retrospective study done at The Royal Gwent Hospital, a District and General Hospital in Wales, UK. One hundred consecutive patients undergoing endourology procedures were recruited retrospectively into the study. This covered the period from July to September 2025.

The procedures included were prostate surgeries, including transurethral resection of prostate (TURP) and holmium laser enucleation of the prostate (HoLEP), transurethral resection of bladder tumours (TURBT), and stone surgeries such as percutaneous nephrolithotomy (PCNL) and ureteroscopy (URS). Patient data was gathered from the electronic medical records system.

Being an observational study, no attempt was made to modify the existing practice. Patient demographics, planned procedure, method of urine testing and its timing (urine dipstick vs urine culture) were looked at. The usual practice involves sending a urine culture during the preoperative assessment visit. All patients received a single intraoperative antibiotic in the theatre, at induction; this is usually prophylactic intravenous gentamicin, unless a patient's allergy prompts otherwise. The use of antibiotics in addition to the intraoperative single dose (and the reason behind the decision) was looked at. Patients were followed up for a period of two weeks to look for any septic complications prompting prolonged hospital stay or readmission after discharge. The diagnosis of septic complications was made by the clinician assessing the patient, and was based on clinical symptoms and laboratory investigations. All patients with presumed infections have cultures sent prior to starting antibiotics, as part of the institution's protocol, but a positive culture was not considered mandatory for classifying a postoperative infection. 

## Results

One hundred consecutive patients undergoing elective endourology procedures were looked at retrospectively. The period covered was from July to September 2025.

There were 67 males and 33 females, with most patients above 60 years of age (72/100). Sixty patients underwent procedures on the lower urinary tract (HoLEP/TURP (25/100) and TURBT (35/100)), and 40 patients underwent upper tract surgeries (URS/PCNL). 

Of the 100 patients, only 15 had urine cultures done in the 2 weeks as stipulated by the guideline. Of the remaining 85 patients, 6 patients had no cultures in the system at all, and 10 patients had cultures done more than a year prior to surgery. The most common organisms isolated were *Escherichia coli *and Klebsiella.

Six of the 100 patients had positive preoperative cultures. Three patients received preoperative antibiotic treatment. Three patients received a course of antibiotics postoperatively as prophylaxis. One patient received both pre- and postoperative antibiotics. One patient received just the one-off antibiotic at induction.

Twelve of the hundred patients received prophylactic antibiotics post-procedure. Of these, three were patients undergoing ureteroscopy and had positive urine cultures. Two patients were known to have recurrent UTIs, while seven did not have a documented indication for the antibiotic. 

Seven patients went on to develop symptomatic UTIs after the operations. Of these, one patient had PCNL and the others had lower tract surgeries. None of these patients had positive pre-op cultures. Most of them presented with abdominal pain/feeling unwell. None of the patients became systemically unwell. Urine cultures were sent for all patients at the time of presumed postoperative UTI, of which one showed mixed growth and one grew *Staphylococcus*. The rest of the urine cultures were negative.

## Discussion

Asymptomatic bacteriuria is defined as the presence of positive urine cultures in the absence of any symptoms of a urinary tract infection (UTI). It is common in the general population, with estimates being upto 5% in healthy premenopausal women and going up to 40-80% in those aged >80 years. Women tend to have a greater chance of ASB irrespective of age group. Foreign bodies, like catheters, stones and stents, can increase the incidence of ASB [1]. It is estimated that 100% of patients with indwelling catheters develop ASB in four weeks of catheterisation [2]. This is not something that requires routine antibiotic treatment. However, in pregnant women and patients undergoing invasive urology procedures, it is recommended that ASB be treated. The optimum duration of the antibiotic course and when exactly the treatment should commence is a matter of controversy [1].

Most urology centres test urine prior to any elective procedures. This is either by a urine dipstick examination or by formal urine cultures. Positive urine tests lead to the last-minute cancellation of elective surgeries. This is a waste of theatre time as well as a preventable issue [4], and so GIRFT undertook an examination of practice and made some recommendations for how to test, what patients to test and what to do about positive results. This was published in April 2025 [5].

The guideline laid down recommendations for what procedures need urine testing, when to test and how to manage positive urine cultures. A urine dipstick is considered inferior, and formal midstream urine cultures are recommended. This should be done 2 weeks before planned surgery, and ASB should be treated with a 48-hour course of antibiotic preop and targeted prophylaxis intraop. Procedures where this is recommended include TURP, TURBT, HoLEP, URS and PCNL. Our study has noted gaps in the urine testing practices in our hospital.

The understanding is that ASB predisposes to urosepsis post op and hence the need for its treatment before operating. However, there is not enough evidence to support this. A Swedish study in 2017 compared patients with treated and untreated ASB undergoing urology surgery and showed that there is no difference in postoperative infection rates when a single preoperative antibiotic injection was administered at the induction of anaesthesia [6]. This included patients undergoing both upper tract and lower tract procedures, done over a period spanning 2 years and recruited over 2000 patients. These findings were mirrored by a similar prospective study from 2019 [7].

There is, however, the question of upper tract vs lower urinary tract surgery. Whether an obstructed system harbouring organisms would be at a higher risk for disseminated sepsis, and whether stones themselves harbour organisms or the renal parenchyma itself. Studies looking specifically at upper tract stone surgeries have shown that it is safe to proceed with surgery without waiting for negative cultures if the patient has received a culture-specific antibiotic course [8]. Stone cultures are more likely to predict postoperative systemic inflammatory response syndrome (SIRS) and sepsis. When protocols advocate treatment of ASB preop, there is no practice of repeating a culture to ensure it is negative prior to operating. A 2024 study from China demonstrated that preoperative positive cultures, along with positive nitrites and leucocytes on urine dip, are all predictors for sepsis following upper tract stone surgery [9]. This, however, does not explain if treating the preoperative cultures eliminates the risk of post-procedure complications. A multicentre study, though, demonstrated that ASB preop is predictive of postoperative infection, but could not demonstrate that treating the same eliminates the risk [10].

It is interesting to note that the patients who developed symptomatic UTIs among the study population did not have positive preoperative urine cultures. This is an important finding, as it highlights the need for looking at other confounding factors that could potentially be contributing to post-procedure infections. It also should alert us to the fact that focusing on preoperative urine cultures alone is not the solution to ensuring better post-procedure outcomes. Patients undergoing upper tract surgeries were given a prophylactic course of antibiotics post-procedure, anticipating an increased risk of serious infections post op. This was based on the patient's history of having recurrent UTIs (2/11) and clinical judgement by the operating surgeon (7/11). This practice is based on the clinical experience of the operating surgeon and possibly represents a group of patients who perhaps would have gone on to develop postoperative infections had it not been anticipated and managed by the responsible clinician. However, this is a lapse when viewed from the perspective of antibiotic stewardship. 

The retrospective and observational nature of this study restricts the statistical significance of our results. Also, other confounding factors like indwelling stents, catheters, stones, comorbidities, etc., which could contribute to infections, need more study. There is non-uniformity in urine testing practice in the trust, and this was discussed and examined in close detail following the interpretation of the results of this study. Measures have been put in place to address this pitfall. The practice of basing antibiotic prescription on clinician preference rather than the antimicrobial guidelines has also contributed to the skewing of results. Perhaps an interventional study design, with clearly laid down guidelines for antibiotic use, might uncover more post-procedure septic complications. Again, the study based the diagnosis of post-procedure infections and initiation of antibiotics on the decision of the clinician managing the patient rather than objective measures like cultures, SIRS or inflammatory markers. This could have resulted in the skewing of results.

In spite of the many limitations, our study managed to bring to our attention the gaps in practice regarding preoperative urine testing in a secondary care hospital. It showed us aspects that could be improved upon. It also made us think about whether all asymptomatic bacteriuria needs to be treated prior to elective endourology procedures. It gave us scope for designing and planning a study with an interventional design to ensure the optimum use of preoperative urine testing and prophylactic antibiotics to ensure better patient outcomes.

## Conclusions

The practice of routine urine testing prior to elective endourology procedures needs revisiting in the era of emerging antibiotic resistance. Judicious intraoperative use of antibiotic prophylaxis and identifying the subset of patients most likely to benefit from targeted therapy is the need of the hour. Our study, though limited by being a single-centre, retrospective, observational study, with a small sample size, has highlighted the importance of further research into this and a multidisciplinary approach to drawing up new guidelines for better patient outcomes.
